# Somatic mutations in leukocytes infiltrating primary breast cancers

**DOI:** 10.1038/npjbcancer.2015.5

**Published:** 2015-06-10

**Authors:** Maria Kleppe, Elizabeth Comen, Hannah Y Wen, Lennart Bastian, Brian Blum, Franck T Rapaport, Matthew Keller, Zvika Granot, Nicolas Socci, Agnès Viale, Daoqi You, Robert Benezra, Britta Weigelt, Edi Brogi, Michael F Berger, Jorge S Reis-Filho, Ross L Levine, Larry Norton

**Affiliations:** 1 Human Oncology and Pathogenesis Program, Memorial Sloan Kettering Cancer Center, New York, NY, USA; 2 Breast Cancer Service, Department of Medicine, Memorial Sloan Kettering Cancer Center, New York, NY, USA; 3 Department of Pathology, Memorial Sloan Kettering Cancer Center, New York, NY, USA; 4 The Bioinformatics Core, Memorial Sloan Kettering Cancer Center, New York, NY, USA; 5 Cancer Biology and Genetics Program, Memorial Sloan Kettering Cancer Center, New York, NY, USA; 6 The Genomics Core, Memorial Sloan Kettering Cancer Center, New York, NY, USA; 7 Leukemia Service, Department of Medicine, Memorial Sloan Kettering Cancer Center, New York, NY, USA

## Abstract

**Background:**

Malignant transformation requires the interaction of cancer cells with their microenvironment, including infiltrating leukocytes. However, somatic mutational studies have focused on alterations in cancer cells, assuming that the microenvironment is genetically normal. Because we hypothesized that this might not be a valid assumption, we performed exome sequencing and targeted sequencing to investigate for the presence of pathogenic mutations in tumor-associated leukocytes in breast cancers.

**Methods:**

We used targeted sequencing and exome sequencing to evaluate the presence of mutations in sorted tumor-infiltrating CD45-positive cells from primary untreated breast cancers. We used high-depth sequencing to determine the presence/absence of the mutations we identified in breast cancer-infiltrating leukocytes in purified tumor cells and in circulating blood cells.

**Results:**

Capture-based sequencing of 15 paired tumor-infiltrating leukocytes and matched germline DNA identified variants in known cancer genes in all 15 primary breast cancer patients in our cohort. We validated the presence of mutations identified by targeted sequencing in infiltrating leukocytes through orthogonal exome sequencing. Ten patients harbored alterations previously reported as somatically acquired variants, including in known leukemia genes (*DNTM3A*, *TET2*, and *BCOR)*. One of the mutations observed in the tumor-infiltrating leukocytes was also detected in the circulating leukocytes of the same patients at a lower allele frequency than observed in the tumor-infiltrating cells.

**Conclusions:**

Here we show that somatic mutations, including mutations in known cancer genes, are present in the leukocytes infiltrating a subset of primary breast cancers. This observation allows for the possibility that the cancer cells interact with mutant infiltrating leukocytes, which has many potential clinical implications.

## INTRODUCTION

In the past decade, targeted, whole-exome, and whole-genome sequencing studies have delineated a spectrum of somatic mutations in human malignancies.^1,2^ These include large-scale sequencing studies in breast cancer, which have identified recurrent mutations in genes and pathways that contribute to malignant transformation and to therapeutic response. The general approach to limit high-throughput genomic studies to cancer cells themselves is, however, in contrast to burgeoning evidence that cancer cells interact with their microenvironment, including stromal cell constituents, infiltrating white blood cells, and circulating inflammatory cytokines originating from local and distant sites.^3,4^ The integrity of the genome in these non-cancer cellular elements is therefore germane. Indeed, previous studies have shown that stromal cells found in breast cancers are characterized by site- and cell-type-specific epigenetic alterations, and reports suggesting the presence of somatic mutations in the tumor microenvironment are also on record.^5–7^ This suggests that cells other than the cancer cells themselves can acquire properties that contribute to tumorigenesis. In addition to tissue-specific stromal cells, circulating and tumor-infiltrating leukocytes can mediate primary tumor growth and metastasis.^8,9^ Recent evidence suggests that tumor-associated stromal cells and infiltrating leukocytes function differently than circulating or bone marrow resident hematopoietic cells.^3,10,11^ In particular, several studies have indicated that the content of lymphoid and myeloid cells infiltrating breast cancers correlates with clinical outcome.^12–15^

We recently demonstrated that some older individuals have clinically inapparent, clonal hematopoiesis characterized by recurrent, somatic mutations in *TET2* (ref. 16). Of note, *Tet2* loss in the hematopoietic compartment leads to increased self-renewal and myeloid bias of hematopoietic cells.^17–19^ These data, and recent genomic studies of circulating hematopoietic stem cells from normal volunteers and from The Cancer Genome Atlas patients suggest that morphologically normal hematopoietic cells acquire mutations over time, most commonly in known leukemia disease alleles.^20,21^ The observation that normal individuals may harbor oncogenic mutations in hematopoietic cells and the interaction between epithelial cancer cells and infiltrating leukocytes raises the possibility of clonal selection in infiltrating leukocytes. Hence, we sought to define whether tumor-infiltrating leukocytes in breast cancer would harbor somatic mutations, and whether these would be enriched in the tumor as compared with peripheral blood.

## PATIENTS AND METHODS

### Patient materials

Breast cancer samples were collected from consecutive patients with primary triple-negative breast cancer who underwent surgery at Memorial Sloan Kettering Cancer Center between 2012 and 2013 (Table 1). Patients treated with neoadjuvant chemotherapy were excluded from the study. Non-triple-negative breast cancers showing prominent lymphocytic infiltrate in core biopsies were also included. All specimens were sectioned and processed for routine pathological examination. Hematoxylin and eosin-stained slides were reviewed by breast pathologists to establish the diagnoses. Estrogen receptor, progesterone receptor and human epidermal growth factor receptor 2 (HER2) status was evaluated by immunohistochemistry according to the American Society of Clinical Oncology/College of American Pathologists guidelines.^22,23^ HER2 fluorescence *in situ* hybridization was performed in one case with equivocal results by immunohistochemistry. Evaluation of tumor-infiltrating leukocytes was performed as described by Loi *et al*.^14^ Tumor-infiltrating leukocytes were scored as following: extensive, ⩾50% infiltration of either stromal or intratumoral lymphocytes; moderate=5–10%; minimal, ⩽5%. Buccal swab samples were collected from each patient. Mononuclear cells and granulocytes were isolated from peripheral blood following a standard Ficoll protocol. A detailed description on clinicopathological features of each patient is listed in Table 1.

### Isolation and processing of tumor-infiltrating cells

All patients included in this study gave informed consent. Fresh tumor cells, stromal cells, and tumor-infiltrating leukocytes were dissociated from the primary tumors by scraping the cutting surface 5–10 times with a surgical scalpel blade. Cell material was collected by rinsing the blade in phosphate-buffered saline. Cells were spun down and resuspended in red cell lysis buffer to remove red blood cells prior to staining with an anti-human CD45-PE-Cy7- or CD45-APC-Cy7-conjugated flow antibody in FACS buffer (phosphate-buffered saline supplemented with 2% bovine serum albumin). Cells were stained for 20 min in the dark at room temperature, washed once with FACS buffer, and passed through a filter. 4′,6-diamidino-2-phenylindole was added before sorting to discriminate live and dead cells. CD45-positive cells were then purified using a FACSAriaIII Cell Sorter (MSKCC Flow Core Facility, Memorial Sloan Kettering Cancer Center, New York, NY, USA).

### Laser-capture microdissection of tumor cells

Ten consecutive 8-μm-thick nuclear fast red-stained representative sections of the tumors were subjected to laser-assisted microdissection on a PALM Robot MicroBeam laser microdissection system (Zeiss, Thornwood, NY, USA), as previously described.^24^ First, non-neoplastic cells, including inflammatory cells, stroma, and normal breast, were ablated. We subsequently microdissected only histologically unequivocal neoplastic cells from each sample under a microscope. Tissue was microdissected directly into extraction buffer, and DNA was extracted using the DNeasy Blood and Tissue Kit (Qiagen, Valencia, CA, USA) and quantified with the Qubit Fluorometer (Invitrogen, Life Technologies, Norwalk, CT, USA).

### DNA extraction and whole-genome amplification

DNA was extracted using the QiaAmp DNA kit (Qiagen) following the manufacture’s instructions. Buccal swabs were processed using the QiaAmp DNA Mini kit (Qiagen) following the manufacture’s instructions. The quality of DNA samples was analyzed with the Agilent Bioanalyzer 2100 (Agilent Technologies, Santa Clara, NY, USA). Samples with insufficient amount of DNA (<500 ng) were whole-genome amplified using the REPLI-g Mini kit (Qiagen) prior to further use in downstream applications. QPCR was performed to assess the quality of WGA DNA.

### Exome sequencing and targeted capture sequencing

DNA extracted from sorted CD45-positive tumor-infiltrating leukocytes and buccal swabs (Supplementary Table 1) was sheared to an average size of 180±80 bp for exome sequencing. For DNA library preparation, 200–250-bp fragments were selected and subjected to PCR amplification. The library was then hybridized to the Agilent SureSelect Human All Exon Kit (Agilent Technologies) and sequencing was performed on the SOLiD 3plus or SOLiD 4 (Applied Biosystems, Grand Island, NY, USA). Targeted sequencing of tumor-infiltrating leukocytes and matched germline DNA of each patient was performed as previously described.^25^

### PCR and 454 sequencing analysis

Sequence reactions were performed on DNA extracted from mononuclear cells, granulocytes, laser-capture-microdissected tumor cells, and tumor-infiltrating leukocytes. All PCR reactions were performed using amplicon-specific fusion primers. Fusion primers contained next to the template-specific sequence a directional primer at the 5′-end followed by a multiplex identifier for barcode sample identification. Samples from 6–8 different patients were mixed, processed for 454 deep sequencing, and run on a Genome Sequencer FLX instrument (454 Life Sciences, Branford, CT, USA). Data were mapped with BWA MEM (BWA is freely available at http://bio-bwa.sourceforge.net; ver 0.7.4) to the full human genome. Multiple mapping reads (mapping quality scores==0) were removed and then the BAM files were processed for base recalibration using the GATK toolkit (https://www.broadinstitute.org/gatk/; ver 3.1). Mutations were called using GATK HaplotypeCaller, which found only two events. In addition, the read pileups were counted at each of the known mutation sites for each sample to compute the actual depth of both the reference and variant allele, and to compute the non-reference allele frequency for each site.

### Variant detection

Paired-end reads were aligned to the human hg19 genome with BWA 0.6.2-r126 (ref. 26). Local realignment at indel regions and baseQ racalibration was done using the GATK suite version 2.8-1 and following recommendations of its authors.^27^ Variants in the targeted tumor–normal sample pairs were called with MuTect version 1.1.4 (ref. 26). We only considered variants that passed the standard Mutect filters and were not present in the non-somatic databases (non-clinical variants from dbSNP, NHLBI exome sequencing project, and our own internal collection of normal tissues). We focused on variants that we could validate either because they were published and characterized as somatic (in COSMIC) or because they were present in more than 10% of the reads of the corresponding exome sequencing data from the same sample.

## RESULTS

### Targeted sequencing analysis of infiltrating white blood cells

**Figure 1:**
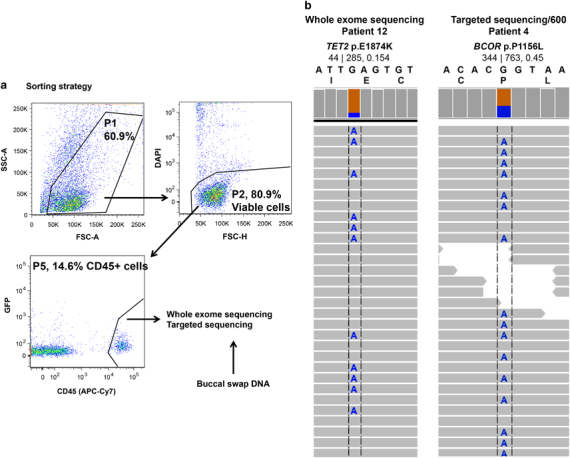
Sequencing analysis of 15 primary breast cancers identified somatically acquired mutations in tumor-infiltrating leukocytes. (**a**) Gating scheme for fluorescent-activated cell sorting of CD45-positive hematopoietic cells. DAPI was included as live-dead stain. Cell doublets were excluded prior to gating on APC-Cy7 (not shown). DNA extracted from the CD45-positive fraction was analyzed using three independent sequencing platforms. (**b**) Representative integrated genomics viewer image showing the presence of acquired mutations. Reads that do not match the reference nucleotide are colored. Gray bar chart on top displays the read depth. Reference nucleotide and protein sequence are depicted for each mutation. Variant allele frequency (VAF) and the number of altered and total reads are shown (alt|total, VAF). DAPI, 4′,6-diamidino-2-phenylindole; GFP, green fluorescent protein.

We obtained fresh samples of 15 untreated primary breast cancers (Table 1) and performed fluorescent-activated cell sorting to separate CD45-positive leukocytes from CD45-negative epithelial cells (Figure 1a). Patients with neoadjuvant chemotherapy were not studied to exclude the effects of chemotherapy on mutational burden. Of the 15 patients, 10 had triple-negative breast cancer, 2 had estrogen receptor-positive disease, HER2-positive disease, 2 had estrogen receptor-positive disease, HER2-negative disease and 1 had estrogen receptor-negative disease, progesterone receptor-positive disease, and HER2-negative disease (Table 1). To obtain coverage for genes with known roles in malignant transformation, we performed capture-based sequencing of 15 paired tumor-infiltrating leukocyte and matched germline (buccal swab) DNA samples (Table 1). We used two capture-based platforms containing all exons of 600 and 341 genes, respectively, and including genes implicated in hematopoietic malignancies and in epithelial malignancies (Supplementary Table 2 and Patients and methods).^25^ Targeted capture of tumor and germline DNA was performed at a mean depth of 384.98±115.27 and 261.50±160.07 (Supplementary Table 1). Variants in the targeted tumor–normal sample pairs were called with MuTect version 1.1.4 (ref. 26). Variants identified by the targeted sequencing platforms that passed the recommended MuTect filters and were not found in any of the somatic databases (non-clinical variants from dbSNP, NHLBI exome sequencing project, and our own internal collection of normal tissue) were annotated as high-confidence variants. This approach identified candidate variants in known cancer genes, including in *BCOR*, *NOTCH2*, *TET2*, *NF1*, *EZH2*, and *JAK1* (Figure 1b). Of importance, mutations in these genes have previously been implicated in the pathogenesis of hematologic malignancies. These data suggest mutations in known cancer genes are present in the white blood cells infiltrating a subset of breast cancers.

### Confirmation of identified variants using exome sequencing

We next performed exome sequencing of tumor-infiltrating leukocytes (mean depth of 118.12±41.29, Supplementary Table 1). We integrated candidate variants identified by the targeted sequencing panels with our exome sequencing data. We considered candidate variants true if more than 10% of the reads presented the alternate allele. Candidate variants identified by capture-based sequencing and previously reported in COSMIC were considered true variants independent of the alternate allele frequency. Following these criteria, we identified a total of 261 somatic mutations (median of 5) in 14 of the 15 patients (93%). Importantly, we identified a total of 47 somatic mutations in 10 of the 15 patients affecting genes commonly mutated in hematological malignancies with an allelic fraction ranging from 2.9 to 51.8% (Table 2; Supplementary Table 3).

### Sequencing analysis of laser-capture-microdissected tumor cells

We next investigated whether the mutations observed in tumor-infiltrating leukocytes were specific to the hematopoietic cells, or whether these alleles were observed in purified breast cancer cells. We performed PCR and high-depth (13,327±6,897, Supplementary Table 4) 454 sequencing on laser-capture-dissected breast cancer cells to look for the presence of specific mutations that we detected in infiltrating white blood cells (Table 2). Importantly, for the microdissection, a system where the laser cuts around the areas selected for microdissection, without damaging the cells of interest, was employed. We identified two *TP53* mutations (patient 2: *TP53* p.R248L and patient 14: *TP53* p.R283P) that were present in purified breast cancer cells, suggesting these mutations originated from the epithelial, malignant clone (Supplementary Table 4). By contrast, all other tested somatic mutations detected in tumor-infiltrating leukocytes, including in known leukemia genes (*DNMT3A*, *TET2*, and *BCOR*, Table 2) were not identified in breast cancer cells consistent with their origin in the leukocyte component (Supplementary Table 4). The two *TET2* mutations were likely pathogenic as we identified a nonsense allele (*TET2* p.Q1702*), which we validated by PCR and sequencing, and a mutation in a highly conserved residue in *TET2* commonly mutated in myeloid malignancies (*TET2* p.E1874K). Mutations in the transcriptional co-repressor *BCOR*, which is targeted by somatic mutations in myeloid leukemia, was identified in one patient.

### Sequencing analysis of circulating leukocytes

Most of the mutations identified in tumor-infiltrating leukocytes and not in breast cancer cells displayed mutant allelic fractions ranging from 5 to 20%. This observation suggests that these mutations were present in enriched subclones and were not rare alleles occurring in a minority of hematopoietic stem cells as previously reported in normal donors. To determine whether the population of tumor-infiltrating leukocytes would be enriched for subclones harboring somatic mutations, we used high-depth (mononuclear cells: 45,640±7,486, granulocytes: 44,891±7,632, Supplementary Table 5) 454 sequencing to look for the presence of the mutations in tumor-infiltrating leukocytes in the circulating leukocytes from these patients. We were able to prospectively obtain peripheral blood samples in compliance with the federal Health Insurance Portability and Accountability Act of 1996 (HIPAA) and Institutional Review Board-approved manner from seven patients in which we had identified somatic mutations in their tumor-infiltrating leukocytes (Table 2). One mutation (patient 2: *DNMT3A* p.Y533C, variant allele frequency: 0.73) was detectable in circulating leukocytes (both mononuclear cells and granulocytes). Of note, the mutation in *DNMT3A* observed in tumor-infiltrating leukocytes and in the peripheral blood was present at 25-fold-higher mutant allele fraction in the tumor-infiltrating leukocytes compared with circulating leukocytes (Supplementary Table 5). The remaining 12 mutations were not detectable by sequencing in circulating leukocytes, likely due to the limits of our sequencing platform. We cannot exclude that these other mutations were present in circulating cells at low allele burden, or alternatively or additionally, in stem/progenitor cells in the bone marrow from these patients. Taken together, these data demonstrate that somatic mutations are highly enriched in tumor-infiltrating leukocytes compared with the overall hematopoietic compartment.

## DISCUSSION

Despite an increasing appreciation of the role of tumor-infiltrating inflammatory cells in solid cancers, studies to date have largely focused on cell non-autonomous interactions between tumor cells and stromal cells, including macrophages, neutrophils, and lymphocytes. In this study, we used high-throughput, next-generation sequencing data to demonstrate that leukocytes with somatic mutations in known cancer genes infiltrate many primary breast cancers. We identified and validated somatic mutations, often affecting known leukemia (*DNTM3A*, *TET2*, and *BCOR)* in tumor-infiltrating leukocytes but not in the cancer cells of 7 of the 15 patients. In one case, the mutation found to be restricted to the tumor-infiltrating leukocytes was also detected in the circulating leukocytes of the same patients, but at a significantly lower frequency. These observations provide direct evidence that at least some cases of primary breast cancer are infiltrated by leukocytes with somatic mutations in genes known to be associated with hematologic malignancies. Notably, a subset of these mutations are in genes that regulate the epigenetic and transcriptional state of hematopoietic cells. Furthermore, it suggests that the leukocytes harboring such somatic mutations can be enriched within the tumor.

Recent studies have highlighted the prognostic role of tumor-infiltrating white blood cells. Studies conducted by Loi *et al.* ^14^ and Adams *et al.* ^15^ recently showed that the quantification of tumor-infiltrating lymphocytes is a prognostic marker for patients with triple-negative breast cancer treated with conventional chemotherapy. To date, studies investigating the prognostic role of infiltrating lymphocytes have not included sequencing data on the population of infiltrating cells. In this respect, our work suggests it is critical to further refine our mutational and functional understanding of infiltrating hematopoietic cells. Increasingly, immunotherapies across multiple types of tumors have focused on harnessing innate immune surveillance against tumor initiation and progression.^28^ The observation that a subset of cancers harbor mutated infiltrating leukocytes may have implications on prognosis and on the response to cytotoxic, targeted, and immune-mediated therapies. It will be important to pursue studies to enumerate functional interactions between breast cancer cells and leukocytes with somatic mutations, through cell–cell contact and/or paracrine secretion of cytokines and chemokines. In addition, it will be important to better delineate the relationship between tumor formation and clonal evolution in infiltrating hematopoietic cells in the setting of immunotherapy. It is not yet known whether the acquisition of mutations within leukocytes impacts tumor initiation and/or progression. If subsequent functional studies demonstrate that clonal leukocytes contribute to cancer progression, this would lead to efforts to target, in parallel, epithelial tumor cells and infiltrating leukocytes in order to increase therapeutic efficacy.

In the previously published studies by Adams *et al.* ^15^ and Loi *et al.* ^14^ of tumor-infiltrating lymphocytes and breast cancer prognosis, the majority of patients received anthracyclines. Secondary hematologic malignancies as a result of chemotherapy, including alkylating agents and anthracyclines, remain a significant risk associated with systemic therapy for epithelial tumors.^29,30^ Our work raises the possibility that some patients may be at increased risk for secondary leukemias based on the presence of oncogenic mutations in infiltrating white cells, which pre-exist before systemic therapy, and which are selected for by cytotoxic chemotherapy. This will need to be examined in prospective studies, and it will be important to determine whether the presence of mutant leukocyte clones at the time of diagnosis should impact therapeutic decisions in different malignant contexts.

Our data resonate with the observations that by the age 70 years, at least 5% of people have known leukemia mutations in a subset of circulating hematopoietic cells.^20^ This includes mutations in the same genes, such as in *DNMT3A*, *TET2*, and *BCOR*, which we identified in tumor-infiltrating leukocytes in our study. However, we identified a much higher proportion of patients with mutations in their tumor-associated leukocytes (93%, 14 out of 15), including in patients at a younger age. These data suggest these mutations are not solely a function of age-related mutations, but rather represent earlier somatic events in patients with breast cancer or tumor-specific enrichment of mutant hematopoietic cells. It will be important to identify whether patients harboring leukemogenic mutations in infiltrating leukocytes are at increased risk for future leukemias, and whether systemic therapies including cytotoxic chemotherapy increase the selective advantage of pre-existing mutant hematopoietic clones. Moreover, it will be important to determine how mutant breast cancer cells might influence the behavior and clonal selection of mutant hematopoietic cells, and vice versa, as their mutual presence may lead to clonal changes in both compartments.

Although our studies identified mutations in purified leukocytes, studies of *en bloc* tumor samples will need to consider the possibility that some mutations detected in tumor biopsies might originate in hematopoietic or other stromal cells and not from the primary tumor or metastasis, in particular studies seeking to identify minor subclones harboring specific mutations or to characterize intratumor genetic heterogeneity. Given that most samples subjected to whole-exome and whole-genome analysis comprises a mixture of cells and stromal cells (e.g., leukocytes, fibroblasts, adjacent normal breast tissue) and that somatic mutations may also be present in leukocytes, if subclonal mutations are identified, defining whether these mutations are present in tumor cells or leukocytes would be required. In addition, subsequent studies will need to delineate which hematopoietic subsets acquire somatic mutations, and whether the functional effects of these disease alleles varies based on the specific hematopoietic context. Future studies with larger patient cohorts will be needed to delineate which tumor-infiltrating hematopoietic cells are most commonly characterized by somatic mutations, and to determine whether mutations in specific hematopoietic subsets can impact tumor growth and therapeutic response. In conclusion, the identification of somatic mutations in breast cancer tumor-infiltrating leukocytes challenges the current paradigm that although aberrant, the tumor microenvironment would not be targeted by clonal somatic alterations. The clinical implications regarding carcinogenesis, clinical course, and response to treatment of these findings warrants further study. These studies will likely reveal novel opportunities for cancer prevention, detection, prognostication, and the development of therapies, which may be directed not only to cancer cells, but also to cells from the microenvironment harboring somatic genetic alterations.

## Figures and Tables

**Table 1:** Summary of clinicopathological features

*ID*	*Age (years)*	*Type*	*Level of lymphocytic infiltration* a	*CD45 (%)*	*Size (cm)*	*HG*	*NG*	*Mitosis*	*OG*	*LVI*	*LN*	*ER*	*PR*	*HER2*	*FISH*
1	40	IDC NOS	Moderate	36.6	2	3	3	3	3	No	No	0	0	0	—
2	72	IDC NOS	Moderate	11.89	1.5	3	3	3	3	No	No	0	0	0	—
3^b^	37	IDC NOS	Extensive	12.5	4.5	3	3	3	3	No	No	<1%	<1%	1+ to 2+	1.3
4	35	IDC NOS	Moderate	0.4	5	2	3	3	3	Yes	Yes	95%	90%	3+	—
5	64	ILC (C/P)	Minimal	5.0	1	3	3	1	2	No	No	99%	10%	1+	—
6	62	Apocrine	Moderate	0.6	3.3	2	3	2	2	Yes	Yes	0	0	0	—
7	83	IDC NOS	Moderate	1.4	3.1	3	3	2	3	Yes	No	0	0	0	—
8	35	IDC NOS	Moderate	19.1	2.3	3	3	3	3	No	No	0	0	0	—
9	39	IDC NOS	Extensive	40.95	3	3	3	3	3	No	No	0	0	1+	—
10	62	IDC NOS	Moderate	7.6	1.8	3	3	3	3	Yes	No	0	0	1+	—
11^c^	53	IDC NOS	Minimal	0.7	1.9	3	3	2	3	No	NA	0	0	1+	—
12	88	Mucinous	Moderate	0.3	6.6	2	1	1	1	No	Yes	95%	60%	0	—
13	56	IDC NOS	Moderate	1.4	2.5	3	3	3	3	Yes	No	5%	5%	3+	—
14	65	IDC NOS	Minimal	1	2.1	3	3	3	3	Yes	NA	0	5%	1+	—
15	72	IDC NOS	Moderate	3	1.3	3	3	3	3	No	No	0	0	0	—

Abbreviations: ER, estrogen receptor; FISH, fluorescence *in situ* hybridization; HER2, human epidermal growth factor receptor 2; HG, histological grade; IDC, invasive ductal carcinoma; ILC, invasive lobular carcinoma; LN, lymph node involvement; LVI, lymphovascular invasion; NA, not sampled; NG, nuclear grade; NOS, not otherwise specified; PR, progesterone receptor; TIL, tumor-infiltrating lymphocyte; OG, overall grade.

a Scoring criteria for the level of lymphocytic infiltration are defined in PATIENTS AND METHODS.

b Patient with concurrent astrocytoma (WHO III).

c Ipsilateral breast cancer recurrence.

**Table 2:** Somatic mutations in known cancer genes

*Sample*	*Gene*	*Mutation*	*Frequency*
1	*EP300*	p.G1777C	0.06
2	*DNMT3A*	p.Y533C	0.185
3	*EZH2*	p.A483S	0.46
	*TP53*	p.M169I	0.029
4	*BCOR*	p.P1156L	0.49
	*EPHA7*	p.G592S	0.14
	*WT1*	p.T278I	0.11
	*TET2*	p.Q1702*	0.06
	*EGFR*	p.A871E	0.042
5	*ALK*	p.R1209Q	0.21
	*ETV6*	p.P25S	0.038
6	*NOTCH2*	p.P1101T	0.18
	*NF1*	p.Q2434H	0.099
	*SMARCA4*	p.D694E	0.087
12	*TET2*	p.E1874K	0.17

Mutations listed in this table were identified by targeted sequencing with an allele frequency of ⩾10%. Mutations occurring at a lower frequency were included if previously reported in COSMIC.
