# Supplementary material for: Somatic mutations in leukocytes infiltrating primary breast cancers
Source: NPJ Breast Cancer. 2015 Jun 10;1:15005–. doi: 10.1038/npjbcancer.2015.5 (PMC5515194; doi:10.1038/npjbcancer.2015.5)
Supplement: Supplementary Information [file npjbcancer20155-s1.doc]

Title: Somatic mutations in leukocytes infiltrating primary breast cancers

**Authors:** Maria Kleppe^1*^, Elizabeth Comen^2*^, Hannah Y Wen^3^, Lennart Bastian^1^, Brian Blum^2^, Franck T. Rapaport^4^, Matthew Keller^2^, Zvika Granot^5^, Nicolas Socci^4^, Agnès Viale^6^, Daoqi You^6^, Robert Benezra^5^, Britta Weigelt^1,3^, Edi Brogi^3^, Michael F Berger^1,3^, Jorge S Reis-Filho^1,3^, Ross L Levine^1,7^, and Larry Norton^2#^

**Affiliations:**

^1^ Human Oncology and Pathogenesis Program, Memorial Sloan Kettering Cancer Center, New York, NY, USA

^2^ Breast Cancer Service, Department of Medicine, Memorial Sloan Kettering Cancer Center, New York, NY

^3^ Department of Pathology, Memorial Sloan Kettering Cancer Center, New York, NY

^4^ Bioinformatics Core, Memorial Sloan Kettering Cancer Center, New York, NY

^5^ Cancer Biology and Genetics Program, Memorial Sloan Kettering Cancer Center, New York, NY

^6^ Genomics Core, Memorial Sloan Kettering Cancer Center, New York, NY

^7^ Leukemia Service, Memorial Sloan Kettering Cancer Center, New York, NY

**Supplementary Materials**

Table S1. Mean target coverage information

Table S2. Genes targeted by Hem-Capture sequencing panel

Table S3. Variants identified in genes commonly mutated in hematological malignancies

Table S4. Deep sequencing analysis of breast tumor cells

Table S5. Deep sequencing analysis of peripheral blood cells

**Table S1. Mean target coverage information**

|  |  | **Mean target coverage** | | |
| --- | --- | --- | --- | --- |
| **Sample source** | **Sample** | **IMPACT** | **Hem/600** | **Exome** |
| CD45+ cells | 1 | 99.81 | 91.48 | 39.70 |
|  | 2 | 218.98 | 243.69 | 75.16 |
|  | 3 | 358.14 | 392.53 | 66.93 |
|  | 4 | 379.38 | 388.45 | 158.95 |
|  | 5 | 386.30 | 345.25 | 84.95 |
|  | 6 | 263.18 | 258.14 | 89.55 |
|  | 7 | 417.28 | 413.19 | 123.42 |
|  | 8 | 476.70 | 470.42 | 86.71 |
|  | 9 | 375.74 | 388.05 | 120.25 |
|  | 10 | 484.63 | 472.99 | 145.36 |
|  | 11 | 319.04 | 411.89 | 150.99 |
|  | 12 | 457.35 | 530.77 | 176.98 |
|  | 13 | 442.33 | 506.16 | 142.88 |
|  | 14 | 459.61 | 550.16 | 150.23 |
|  | 15 | 431.70 | 515.91 | 159.63 |
| Germline control | 1 | 219.87 | 354.11 | 110.69 |
|  | 2 | 68.86 | 127.06 | -^1^ |
|  | 3 | 228.57 | 383.17 | 138.63 |
|  | 4 | 182.19 | 273.96 | -^1^ |
|  | 5 | 260.48 | 377.91 | 136.01 |
|  | 6 | 333.29 | 449.72 | -^1^ |
|  | 7 | 59.82 | 28.49 | -^1^ |
|  | 8 | 262.09 | 326.85 | 124.31 |
|  | 9 | 176.59 | 242.46 | -^1^ |
|  | 10 | 283.65 | 274.74 | -^1^ |
|  | 11 | 321.25 | 706.71 | -^1^ |
|  | 12^2^ | 2.49 | 3.76 | -^1^ |
|  | 13 | 262.84 | 479.45 | 115.57 |
|  | 14 | 112.34 | 205.98 | -^1^ |
|  | 15 | 283.08 | 553.12 | -^1^ |

^1^Samples were not run at the indicated sequencing platform. ^2^CD45-positive sample of patient #12 was compared against pooled buccal swab samples due to low coverage of the matching germline DNA samples.

**Table S2. Genes targeted by Hem-Capture sequencing panel**

**Table S3. Variants identified in genes commonly mutated in hematological malignancies**

| **Sample** | **Gene** | **Mutation** | **Chr** | **Position** | **Ref** | **Alt** | **IMPACT** | **Hem** | **Exome**  **(alt | tot)** |
| --- | --- | --- | --- | --- | --- | --- | --- | --- | --- |
| 1 | ***EP300*** | **p.G1777C**^1^ | 22 | 41573044 | G | T | 0.024 | 0.06 | 9 | 25 |
| 2 | *MPL* | p.E54V | 1 | 43803851 | A | T | 0.15 | 0.143 | 17 | 88 |
|  | *FLT3* | p.Q394* | 13 | 28622437 | G | A | 0.14 | 0.156 | 5 \| 29 |
|  | *JAK1* | p.S260G | 1 | 65332761 | T | C | 0.17 | 0.154 | 14 \| 108 |
|  | *MLL* | p.P1843S | 11 | 118366587 | C | T | 0.09 | 0.08 | 3 \| 25 |
|  | ***TP53***^2^ | **p.R248L** | 17 | 7577538 | C | A | 0.086 | 0.086 | 2 \| 42 |
|  | ***DNMT3A*** | **p.Y533C** | 2 | 25467478 | T | C | 0.18 | 0.185 | 0 \| 5 |
| 3 | *EZH2* | p.A478S | 7 | 148513834 | C | A | 0.18 | 0.259 | 12 | 25 |
|  | *MLL2* | p.R171_splice | 12 | 49447925 | T | A | 0.31 | 0.314 | 72 \| 182 |
|  | *EP300* | p.Q2355L | 22 | 41574779 | A | T | 0.27 | 0.258 | 93 \| 276 |
|  | *EP300* | p.M1972T | 22 | 41573630 | T | C | 0.29 | 0.287 | 55 \| 230 |
|  | *SMC3* | p.E251K | 10 | 112342347 | G | A | not targeted | 0.192 | 11 \| 74 |
|  | *APC* | p.Q2701H | 5 | 112179394 | G | T | 0.069 | not targeted | 3 \| 29 |
|  | *CSF3R* | p.L18R | 1 | 36945045 | A | C | not targeted | 0.041 | 9 \| 89 |
|  | ***SETBP1*** | **p.K469Q** | 18 | 42530710 | A | C | not targeted | 0.055 | 11 \| 143 |
|  | ***TP53*** | **p.M169I** | 17 | 7578423 | C | T | 0.029 | 0.024 | 4 \| 252 |
| 4 | ***BCOR*** | **p.P1156L**^1^ | X | 39923624 | G | A | 0.49 | 0.451 | 38 | 84 |
|  | *MLL* | p.A2061T | 11 | 118371733 | G | A | 0.17 | 0.157 | 51 \| 262 |
|  | *SOCS1* | p.C42S | 16 | 11349211 | C | G | 0.066 | 0.091 | 3 \| 24 |
|  | ***WT1*** | **p.T278I**^1^ | 11 | 32449541 | G | A | 0.11 | 0.112 | 17 \| 142 |
|  | *ABCA1* | p.T1750I | 9 | 107558467 | G | A | not targeted | 0.091 | 21 \| 184 |
|  | ***TET2*** | **p.Q1702***^1^ | 4 | 106196771 | C | T | 0.06 | 0.054 | 8 \| 137 |
|  | ***PTEN*** | **p.A126V** | 10 | 89692893 | C | T | - | 0.034 | 2 \| 38 |
|  | ***BPTF*** | **p.E234D** | 17 | 65850144 | G | T | not targeted | 0.037 | 0 \| 49 |
| 5 | *BPTF* | p.G2697R | 17 | 65955819 | G | C | not targeted | 0.191 | 4 | 17 |
|  | *JAK3* | p.Q1094* | 19 | 17937647 | G | A | 0.23 | 0.25 | 58 \| 257 |
|  | *MLL2* | p.H5200L | 12 | 49420150 | T | A | 0.18 | 0.149 | 15 \| 79 |
|  | *ABCA1* | p.L39Q | 9 | 107651427 | A | T | not targeted | 0.037 | 1 \| 6 |
|  | *EP300* | p.S692C | 22 | 41542763 | A | T | 0.085 | 0.107 | 17 \| 129 |
|  | *DICER1* | p.H1652Y | 14 | 95562303 | G | A | 0.097 | not targeted | 27 \| 228 |
|  | *ASXL1* | p.G792D | 20 | 31022890 | G | A | 0.18 | 0.164 | 1 \| 9 |
|  | ***ETV6*** | **p.P25S**^1^ | 12 | 11905423 | C | T | 0.031 | 0.038 | 10 \| 264 |
|  | ***SF3B1*** | **p.N619K** | 2 | 198267500 | G | T | - | 0.056 | 0 \| 20 |
| 6 | ***EP300*** | **p.R1737H**^1^ | 22 | 41572925 | G | A | 0.13 | 0.105 | 9 | 42 |
|  | *STAG2* | p.L364P | X | 123185044 | T | C | 0.045 | 0.038 | 3 \| 15 |
|  | *MLL* | p.K3846M | 11 | 118392035 | A | T | 0.32 | 0.2356 | 4 \| 20 |
|  | *APC* | p.E243_splice | 5 | 112128227 | G | T | 0.16 | not targeted | 4 \| 22 |
|  | *KIT* | p.G93S | 4 | 55561887 | G | A | 0.12 | 0.129 | 6 \| 46 |
|  | *MLL2* | p.G4840E | 12 | 49421710 | C | T | 0.038 | 0.035 | 20 \| 159 |
|  | *MLL2* | p.S678P | 12 | 49445434 | A | G | - | 0.053 | 3 \| 26 |
|  | ***PTEN*** | **p.A3T** | 10 | 89624233 | G | A | 0.17 | - | 3 \| 28 |
|  | ***KRAS*** | **p.A155V** | 12 | 25368481 | G | A | - | 0.035 | 2 \| 71 |
| 7 | ***CTNNA1*** | **p.R451*** | 5 | 138240092 | C | T | not targeted | 0.518 | 50 | 95 |
| 12 | *TET2* | p.E1874K | 4 | 106197287 | G | A | 0.17 | 0.138 | 44 | 285 |
| 14 | ***TP53***^2^ | **p.R283P** | 17 | 7577090 | C | G | 0.055 | 0.065 | 5 | 88 |
|  | ***MLL2*** | **p.Q3264K** | 12 | 49431349 | G | T | - | 0.046 | 0 \| 52 |
| 15 | ***NOTCH1*** | **p.R1279S** | 9 | 139401234 | G | T | - | 0.06 | 0 | 31 |

Variants highlighted in bold were previously described in COSMIC. ^1^Indicates variants altering a codon previously reported in COSMIC, but result in a different substitution of the same amino acid. ^2^454 sequencing showed that mutations were present in purified breast cancer cells. Not targeted, specific gene not targeted by respective sequencing platform. Ref, reference nucleotide; alt, altered nucleotide; chr, chromosome. Data from three sequencing platforms (Hem-Capture panel (Hem), IMPACT, and exome sequencing data) are shown.

**Table S4. Deep sequencing of breast tumor cells**

| **Sample** | **Gene** | **Mutation** | **# variant**  **reads** | **VAF**  **tumor cells**  **[%]** | **Coverage depth** | **VAF**  **tumor-infiltrating leukocytes [%]** |
| --- | --- | --- | --- | --- | --- | --- |
| 1 | *EP300* | p.G1777C | 2 | 0.01 | 19460 | 6.0 |
| 2 | *DNMT3A* | p.Y533C | 1 | 0.01 | 17707 | 18.5 |
|  | *TP53* | p.R248L | 6547 | 71.34 | 9177 | 8.6 |
| 3 | *EZH2* | p.A483S | 0 | 0.00 | 14518 | 46.0 |
|  | *TP53* | p.M169I | 0 | 0.04 | 21792 | 2.9 |
| 4 | *BCOR* | p.P1156L | 4 | 0.05 | 8521 | 49.0 |
|  | *EPHA7* | p.G592S | 1 | 0.01 | 9060 | 14.0 |
|  | *WT1* | p.T278I | -^1^ | -^1^ | N/A | 11.0 |
|  | *TET2* | p.Q1702* | 54 | 0.26 | 20909 | 6.0 |
|  | *EGFR* | p.A871E | 0 | 0.00 | 5844 | 4.2 |
| 5 | *ALK* | p.R1209Q | 14 | 0.15 | 9426 | 21.0 |
|  | *ETV6* | p.P25S | 0 | 0.00 | 3136 | 3.8 |
| 6 | *NOTCH2* | p.P1101T | -^1^ | -^1^ | N/A | 18.0 |
|  | *NF1* | p.Q2434H | 2 | 0.04 | 5361 | 9.9 |
|  | *SMARCA4* | p.D694E | -^1^ | -^1^ | N/A | 8.7 |
| 12 | *TET2* | p.E1874K | 356 | 2.03 | 17567 | 17.0 |
| 14 | *TP53* | p.R283P | 21327 | 88.48 | 24104 | 6.5 |

VAF, variant allele frequency; ^1^not sequenced; N/A, not applicable.

**Table S5. Deep sequencing of peripheral blood cells from breast cancer patients**

| **Sample** | **Gene** | **Mutation** | **# variant**  **reads** | **VAF**  **MNC**  **[%]** | **Coverage depth** | | **# variant**  **reads** | | **VAF**  **Granulocytes [%]** | **Coverage**  **depth** | **VAF**  **tumor-infiltrating leukocytes [%]** |
| --- | --- | --- | --- | --- | --- | --- | --- | --- | --- | --- | --- |
| 1 | *EP300* | p.G1777C | 3 | 0.01 | | 44363 | | 3 | 0.01 | 45130 | 6.0 |
| 2 | *DNMT3A* | p.Y533C | 363 | 0.73 | | 50062 | | 669 | 1.34 | 50046 | 18.5 |
| 3 | *EZH2* | p.A483S | 0 | 0.00 | | 30057 | | 0 | 0.00 | 23722 | 46.0 |
|  | *TP53* | p.M169I | 7 | 0.02 | | 41638 | | 4 | 0.01 | 38776 | 2.9 |
| 4 | *BCOR* | p.P1156L | 19 | 0.04 | | 50003 | | 10 | 0.02 | 49992 | 49.0 |
|  | *EPHA7* | p.G592S | 10 | 0.02 | | 50063 | | 7 | 0.01 | 50045 | 14.0 |
|  | *WT1* | p.T278I | 13 | 0.03 | | 47802 | | 14 | 0.03 | 41982 | 11.0 |
|  | *TET2* | p.Q1702* | 20 | 0.04 | | 49539 | | 29 | 0.07 | 41051 | 6.0 |
|  | *EGFR* | p.A871E | 0 | 0.00 | | 50069 | | 1 | 0.00 | 50076 | 4.2 |
| 5 | *ALK* | p.R1209Q | 18 | 0.04 | | 49999 | | 20 | 0.04 | 49991 | 21.0 |
|  | *ETV6* | p.P25S | 18 | 0.06 | | 29627 | | 33 | 0.08 | 42677 | 3.8 |
| 6 | *NOTCH2*^1^ | p.P1101T | - | - | | N/A | | - | - | N/A | 18.0 |
|  | *NF1* | p.Q2434H | 0 | 0.00 | | 50039 | | 0 | 0.00 | 50040 | 9.9 |
|  | *SMARCA4* | p.D694E | 1 | 0.00 | | 50057 | | 0 | 0.00 | 50051 | 8.7 |
| 12 | *TET2*^1^ | p.E1874K | - | - | | N/A | | - | - | N/A | 17.0 |

PB, peripheral blood; MNC, mononuclear cells, depth, number of total reads; N/A, not applicable. ^1^not sequenced.
